# Roles of plant hormones in thermomorphogenesis

**DOI:** 10.1007/s44154-021-00022-1

**Published:** 2021-12-18

**Authors:** Hai-Ping Lu, Jing-Jing Wang, Mei-Jing Wang, Jian-Xiang Liu

**Affiliations:** State Key Laboratory of Plant Physiology and Biochemistry, College of Life Sciences, Zhejiang University, Hangzhou, 310027 China

**Keywords:** Arabidopsis, Auxin, BR, Ethylene, GA, JA, Temperature, Thermomorphogenesis

## Abstract

Global warming has great impacts on plant growth and development, as well as ecological distribution. Plants constantly perceive environmental temperatures and adjust their growth and development programs accordingly to cope with the environment under non-lethal warm temperature conditions. Plant hormones are endogenous bioactive chemicals that play central roles in plant growth, developmental, and responses to biotic and abiotic stresses. In this review, we summarize the important roles of plant hormones, including auxin, brassinosteroids (BRs), Gibberellins (GAs), ethylene (ET), and jasmonates (JAs), in regulating plant growth under warm temperature conditions. This provides a picture on how plants sense and transduce the warm temperature signals to regulate downstream gene expression for controlling plant growth under warm temperature conditions via hormone biosynthesis and signaling pathways.

## Introduction

Plants constantly adjust their growth and developmental programmes in accordance with environmental temperatures (Legris et al. 2017; Vu et al. 2019a). With the global warming effects, such adaptations are quite important for ensuring plant survival, productivity, and ecological distribution (Xu et al. 2020; Sun et al. 2020). Even a modest shift in environmental growth temperature can significantly alter the expression of hundreds of temperature responsive genes, leading to obvious morphological and developmental changes, including hypocotyl and petiole elongation, leaf hyponasty, stomata movement and accelerated flowering, in a process collectively referred to as thermomorphogenesis (Quint et al. 2016; Casal and Balasubramanian 2019; Vu et al. 2019b). The term thermomorphogenesis is analogy to photomorphogenesis (light-mediated growth), to describe the effects of environmental temperature on plant morphology. The process of thermomorphogenesis is similar to skotomorphogenesis (growth in the dark) in terms of hypocotyl elongation, but the underlying mechanisms could be quite different (Delker et al. 2014). Plant thermomorphogenesis has been widely studied in recent years in view of its climatic association with global warming, a worldwide ecological and agricultural concern. Because increases in global temperature are expected to dramatically reduce crop productivity (Schlenker and Roberts 2009; Nicotra et al. 2010; Rathore et al. 2020), understanding the mechanism of warm temperature responses has become imminent for knowledge-based crop designs to sustain crop production in a changing climate.

Various signaling mediators and associated signaling mechanisms have been revealed in plant responses to warm temperatures (Zhang et al. 2021b). Among them, the PHYTOCHROME INTERACTING FACTOR 4 (PIF4) acts as a central transcription regulator (Vu et al. 2019b), which is regulated at both the transcriptional and protein levels (Park et al. 2017; Gangappa and Kumar 2017; Han et al. 2019; Zhang et al. 2017). PIF4 induces the transcription of downstream genes and promotes organ elongation and flowering under warm temperature conditions (Franklin et al. 2011; Sun et al. 2012; Kumar et al. 2012).

Plant hormones, or phytohormones, are endogenous small chemical molecules that play diverse physiological roles in plant growth, development, and environmental stress and defense responses (Verma et al. 2016; Depuydt and Hardtke 2011). These includes seven classical plant hormones, auxin, cytokinin (CK), Gibberellins (GAs), ethylene (ET), abscisic acid (ABA), brassinosteroids (BRs) and Strigolactone (SL) (Peres et al. 2019; Burger and Chory 2020; Binder 2020; Chen et al. 2020; Shu et al. 2018; Cortleven et al. 2019; Zhao 2018). In this review, we summarize and discuss recent research progress on how thermomorphogenic growth is regulated in plants and highlight the important role of phytohormones including auxin, BRs, GAs, and ET in plant thermomorphogenesis. Hypocotyl elongation is one of the earliest phenotypes in plant response to warm temperature, therefore we mainly focused on the shoot thermomorphogenesis in the current paper.

### Warm temperature sensing

**Fig. 1:**
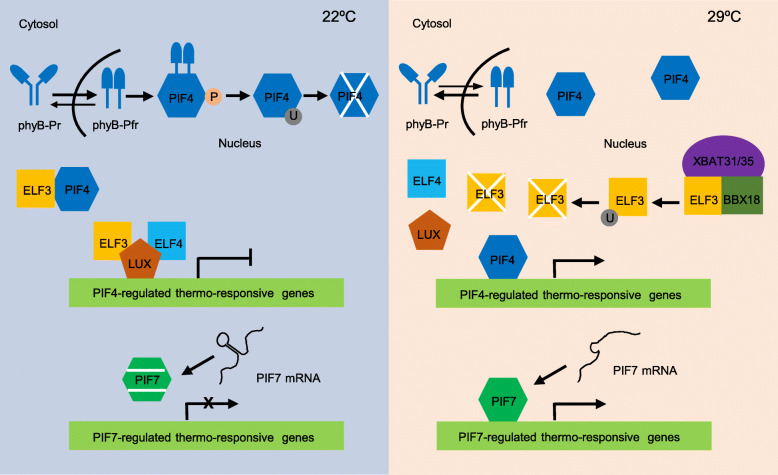
An overview of the signal transduction and gene regulation in plant thermomorphogenesis. phyB, ELF3 and PIF7 are proposed thermosensors. Both PIF4 and PIF7 are key transcriptional regulators of downstream genes involved in hypocotyl growth. At ambient temperatures (22 °C), photoreceptor phyB readily converts from the inactive form Pfr to the active form Pfr, and phyB-Pfr binds to PIF4, leading to PIF4 phosphorylation and subsequently proteasome-mediated degradation; ELF3 functions as a negative regulator of PIF4 by inhibiting the expression of *PIF4* together with ELF4 and LUX in the evening complex (EC), and by inhibiting the protein activity of PIF4 independent on EC; the secondary structure of *PIF7* mRNA also inhibits the protein translation of PIF7. At elevated warm temperatures (29 °C), the conversation of phyB from Pfr back to Pr is accelerated, therefore releasing the inhibitory effects of phyB on PIF4; ELF3 is degraded by the E3 ubiquitin ligase XBAT31/35 together with BBX18, diminishing the inhibitory effect of ELF3 on PIF4; *PIF7* mRNA has a conformation change, which enhances PIF7 translation. More PIF4 and PIF7 activities result in faster hypocotyl elongation in Arabidopsis. BBX18, B-box 18; ELF3/4, EARLY FLOWERING 3/4; LUX, LUX ARRHYTHMO; PHYB, phytochrome B; PIF4/7, phytochrome interacting factor 4/7; XBAT31/35, XB3 ORTHOLOG 1/5 IN *Arabidopsis thaliana*

Plants precisely sense environmental temperature changes to adjust their growth and development. Multiple warm temperature sensors and thermosensory mechanisms in plants have been identified (Fig. 1). For example, phytochrome B (phyB) was identified as a thermosensor which also acts as a photoreceptor (Legris et al. 2016; Jung et al. 2016). It exists as two interconvertible photochemical forms in plants: the red light-absorbing Pr and the far-red light-absorbing Pfr (Burgie and Vierstra 2014). The photo-activated Pfr of phyB thermodynamically reverts to the Pr form in darkness, which is termed ‘dark reversion’ or ‘thermal conversion’ (Klose et al. 2020). Notably, warm temperatures accelerate the Pfr-to-Pr dark reversion, releasing the inhibitory effect of phyB on PIF4 (Legris et al. 2016; Jung et al. 2016). In addition, the number of phyB-containing photobodies decreases under warm temperature conditions (Hahm et al. 2020), demonstrating a thermosensory mechanism controlled by phyB. The rate of thermal reversion of phyB, in particular, is rapid enough to rival that of photoactivation and accelerates with temperature increases of between 10 °C and 30 °C (Legris et al. 2016). These intrinsic properties of the phyB molecule enable the activity of phyB to respond to changes in environmental temperatures (Fig. 1).

Early Flowering 3 (ELF3) is a recently proposed thermosensor (Jung et al. 2020). It is also an essential component in the Evening Complex (EC) of plant circadian clock (Nusinow et al. 2011). The ELF3 protein has a prion-like domain (PrD) with embedded polyglutamine (polyQ) repeat, which is important for inducing the formation of protein condensates via liquid-liquid phase separation (Jung et al. 2020). ELF3 recruits ELF4 and LUX ARRHYTHMO (LUX) to repress the transcriptional expression of *PIF4* during early night (Nusinow et al. 2011; Thines and Harmon 2010). Therefore, the expression of *PIF4* is rhythmic and tightly regulated by the circadian clock (Nozue et al. 2007; Kunihiro et al. 2011). ELF3 also suppresses the protein activity of PIF4 in an EC-independent manner by preventing PIF4 from activating its transcriptional targets in the light (Nieto et al. 2015). The accumulation of ELF3 is reduced under warm temperature conditions via XB3 ORTHOLOG 1 IN *Arabidopsis thaliana* (XBAT31)- and XBAT35-mediated ubiquitination and degradation with the B-box protein BBX18 acting as a scaffolding protein, releasing the repression effects of ELF3 on PIF4 (Ding et al. 2018; Zhang et al. 2021c; Zhang et al. 2021a) (Fig. 1).

PIF4, a bHLH transcription factor in Arabidopsis, induces the expression of downstream genes to promote organ elongation and flowering (Franklin et al. 2011; Kumar et al. 2012)*. PIF7* is a homologous gene to *PIF4*, but it is recently reported to be involved in warm temperature sensing (Chung et al. 2020). The secondary structure of *PIF7* mRNA has a conformation change in response to warm temperatures, leading to the enhanced translation of PIF7 protein, representing a new type of thermosensing (Chung et al. 2020; Vu et al. 2019a) (Fig. 1). PIF7 also directly promotes thermomorphogenic growth by regulating downstream gene expression alone or together with PIF4 (Fiorucci et al. 2020; Chung et al. 2020).

### Auxin and thermomorphogenesis

Both auxin biosynthesis and auxin signaling are required for PIF4-mediated hypocotyl elongation at warm temperatures (Casal and Balasubramanian 2019). The auxin indole-3-acetic acid (IAA) is synthesized from L-tryptophan, via a main indole-3-pyruvic acid pathway, involving the TRYPTOPHAN AMINOTRANSFERASE OF ARABIDOPSIS (TAA) and YUCCA (YUC) flavin monooxygenase families of enzymes (Table 1) (Won et al. 2011). Free IAA levels in aerial tissues are increased under warm temperature conditions (Gray et al. 1998), which is caused by temperature-mediated binding of PIF4/PIF7 to promoter, and subsequent activation of auxin biosynthesis genes such as *YUC8* (Franklin et al. 2011; Sun et al. 2012; Fiorucci et al. 2020). Polar auxin transport is also important for thermoresponses. High temperature enhances shootward auxin transport in roots by promoting the efficient targeting of PIN-FORMED2 (PIN2) to the plasma membrane. More PIN2 accumulation to the plasma membrane makes it capable of transporting more auxin out of the cell and, thus, of maintaining the optimal cellular auxin concentration to promote root growth and development, which dependents on SORTING NEXIN1 (SNX1) through an endosomal trafficking pathway (Hanzawa et al., 2013). Transcriptomics analysis suggested that a mobile auxin signal connects temperature sensing in cotyledons with growth responses in hypocotyls (Bellstaedt et al. 2019).

**Table 1:** Regulation of phytohormone biosynthesis and catabolism by warm temperatures

Phytohormone	Locus	Name	Enzyme activity	Expression	Regulator	Reference
Auxin biosynthesis	At4g28720	YUC8	Indole-3-pyruvate monooxygenase	up	PIF4	(Sun et al. 2012)
	At4g39950	CYP79B2	Tryptophan N-monooxygenase	up	PIF4	(Franklin et al. 2011)
	At1g70560	TAA1	Tryptophan aminotransferase	up	PIF4	(Franklin et al. 2011)
GA biosynthesis	At4g25420	GA20OX1	Gibberellin 20 oxidase	up	TCP15	(Ferrero et al. 2019)
	At1g15550	GA3OX1	Gibberellin 3 oxidase	up	unknown	(Stavang et al. 2009)
GA inactivation	At1g78440	GA2OX1	Gibberellin 2 oxidase	down	unknown	(Stavang et al. 2009)
BR biosynthesis	At5g05690	CPD/CYP90A1	Steroid C-23 hydroxylase	up	PIF4-BES1	(Martinez et al. 2018)
	At3g50660	DWF4/CYP90B1	Steroid C-22 hydroxylase	up	PIF4-BES1	(Martinez et al. 2018)
	At3g30180	BR6OX2	Brassinosteroid-6-oxidase	up	PIF4-BES1	(Martinez et al. 2018)
JA inactivation	At3g11180	JOX1	Jasmonic acid oxidase	up	unknown	Zhu et al., 2021
	At5g05600	JOX2	Jasmonic acid oxidase	up	unknown	Zhu et al., 2021
	At3g55970	JOX3	Jasmonic acid oxidase	up	unknown	Zhu et al., 2021
	At5g07101	ST2A	Sulfotransferase	up	PIF4	(Ding et al. 2018)

Increased intracellular auxin activates a transcriptional cascade mediated by the TRANSPORT INHIBITOR RESPONSE 1/AUXIN-RELATED F-BOX (TIR1/AFB) family of F-box proteins, which functions as the auxin receptor, and the auxin response factors (ARFs) and AUX/IAA families of transcriptional regulators (Salehin et al. 2015). The ubiquitination of AUX/IAA by the auxin–SCF^TIR1/AFB^ complex targets these negative regulators AUXs/IAAs for proteasomal degradation and relieves their inhibition on ARFs, enabling the expression of downstream gene expression (Salehin et al. 2015). Indeed, auxin responsive genes such as *Small auxin up RNA 19* (*SAUR19*) are induced by warm temperatures (Kim et al. 2020; Ding et al. 2018; Franklin et al. 2011).

### BR and thermomorphogenesis

**Fig. 2:**
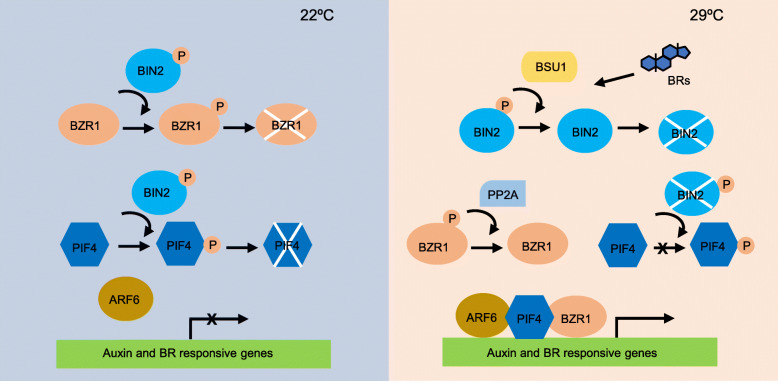
Regulation of plant thermomorphogenesis by the BR signaling pathway. Under ambient temperature (22 °C) conditions, when BR content is low, BZR1 and PIF4 are phosphorylated and inactivated by BIN2. Under warm temperature conditions (29 °C), the phosphatase BSU1 dephosphorylates and inactivates BIN2 when BR signal is perceived, releasing the inhibitory effect of BIN2 on PIF4. Meanwhile, BZR1 is dephosphorylated and activated by a group of PP2A. PIF4 regulates downstream genes for promoting hypocotyl elongation. BZR1 and ARF6 further enhance the transcription activation activity of PIF4 by interacting with PIF4. ARF6, AUXIN RESPONSE FACTOR 6; BIN2, BRASSINOSTEROID INSENSITIVE2; BSU1, *bri1*-SUPPRESSOR1; BRs, brassinosteroids; BZR1, BRASSINAZOLE-RESISTANT 1; PIF4, phytochrome interacting factor 4; PP2A; protein phosphatase2A

BRs are the polyhydroxylated steroidal hormones that are ubiquitously present in the plant kingdom (Peres et al. 2019). BR-mediated thermomorphogenesis is mainly mediated by BR-regulated transcription factor, BRASSINAZOLE RESISTANT1 (BZR1) (Ibanez et al. 2018) (Fig. 2**)**. BZR1 becomes dephosphorylated and activated, presumably through the action of the protein phosphatase2A (PP2A) (Tang et al. 2011). Further protein-DNA binding experiments have demonstrated that BZR1 can both bind the 5′-CGTG(T/C)G-3′ elements (BRRE) and 5′-CANNTG-3′ (E-box) of their target genes, with a preference for BRRE (He et al. 2002; Yin et al. 2002; Sun et al. 2010). Warm temperatures promote the formation of a PIF4-BRI1 EMS SUPPRESSOR 1 (BES1) complex to activate the expression of rate-limiting BR biosynthetic genes (Martinez et al. 2018) (Table 1).

In the normal growth temperature environment, the BR content in plants is relatively low, BZR1 is phosphorylated and inactivated by the cytoplasmic glycogen synthase kinase 3 (GSK3)-like protein kinase BRASSINOSTEROID INSENSITIVE2 (BIN2) (Li and Nam 2002; He et al. 2002; Yin et al. 2002) (Fig. 2**)**. Under warm temperature, BR is accumulated, which inactivates BIN2 by a Ser/Thr phosphatase bri1-SUPPRESSOR1 (BSU1) (Kim et al. 2009), leading to dephosphorylation of BZR1. The dephosphorylated BZR1 regulates gene expression and promotes thermoresponsive hypocotyl elongation. Surprisingly, genome-wide identification of BZR1 and PIF4 binding sites revealed that these two transcription factors share thousands of target genes (Oh et al. 2012). BZR1 and PIF4 cooperatively regulate the expression of co-target genes, including genes involved in cell elongation (Oh et al. 2012). Consistent with their interdependency on the regulation of target gene expression, both BZR1 and PIF4 are required for hypocotyl elongation in warm temperatures (Oh et al. 2012). BZR1 also induces the expression of *PIF4* and other growth promoting genes at elevated temperature in a feedback loop of auxin signaling (Ibanez et al. 2018). In addition to BZR1, the BR-regulated kinase, BIN2, also interacts with and inactivates PIF4 (Bernardo-Garcia et al. 2014). BIN2 phosphorylates PIF4 as well as BZR1, and this phosphorylation marks PIF4 for proteasome-mediated degradation, thereby reducing cellular PIF4 levels (Fig. 2**)**. At the molecular level, auxin and BR regulate the expression of common target genes, which is mediated by a direct interaction between AUXIN RESPONSE FACTOR 6 (ARF6) and BZR1 (Oh et al. 2012). ARF6 also interacts with PIF4, the interactions with BZR1 and PIF4 increase ARF6 activity by enhancing its binding to target promoters (Oh et al. 2014).

### GAs and thermomorphogenesis

GAs are derived from diterpenoids and perceived by a soluble receptor, GIBBERELLIN INSENSITIVE DWARF 1 (GID1), which was originally identified from rice (*Oryza sativa*) and has three redundantly acting homologs GID1A, GID1B, and GID1C in Arabidopsis (Nakajima et al. 2006; Ueguchi-Tanaka et al. 2005). The current model suggests that GA-binding to GID1 receptor leads to conformation changes and nuclear localization of the receptors, which promotes their interaction with the DELLA proteins, targeting DELLA proteins for degradation by the ubiquitin-proteasome pathway mediated by the SLEEPY1 (SLY1) F-box protein (McGinnis et al. 2003; Griffiths et al. 2006; Dill et al. 2004; Willige et al. 2007). DELLAs, including GIBBERELLIC ACID INSENSITIVE (GAI), REPRESSOR OF ga1–3 (RGA), RGA-like 1 (RGL1), RGL2, and RGL3, are transcriptional regulators that restrict plant growth presumably by causing transcriptional reprogramming (Sun 2011). Therefore, GA promotes plant growth by means of removing the inhibitory DELLA proteins.

**Fig. 3:**
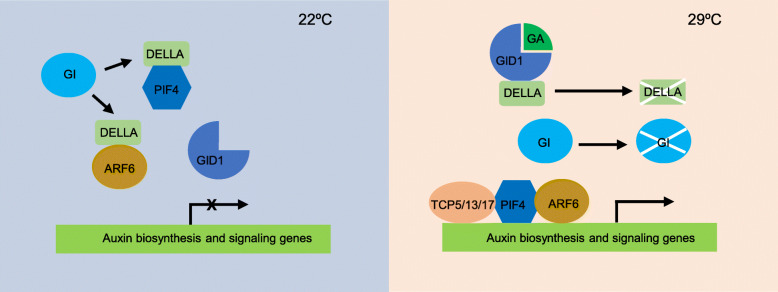
Regulation of plant thermomorphogenesis by the GA signaling pathway. Under ambient temperature (22 °C) conditions, when GA contents is low, DELLA binds to PIF4 and ARF6, blocking the DNA-binding activities of PIF4 and ARF6. Under warm temperature conditions (29 °C), GA content is increased, leading to formation of the GA-GID1-DELLA complex and DELLA degradation, therefore, releasing the inhibitory effects of DELLA on PIF4 and ARF6. PIF4 regulates downstream genes for promoting hypocotyl elongation, which is enhanced by ARF6 and TCP5/13/17. Further, GI binds and stabilizes the DELLA protein RGA under long day condition. Under short day condition at warm temperature, GI is degraded at high level of GA, therefore, the inhibitory effect of DELLA is released and the hypocotyl length is longer under short day condition than that under long day condition at warm temperature. ARF6, AUXIN RESPONSE FACTOR 6; GA, gibberellin; GI, GIGANTEA; GID1, GIBBERELLIN INSENSITIVE DWARF1; PIF4, phytochrome interacting factor 4; TCP5/13/17, TEOSINTE BRANCHED 1, CYCLOIDEA, PCF 5/13/17

Previous studies demonstrated that GA is required for temperature-mediated hypocotyl elongation (Stavang et al. 2009), and the lack of the DELLA protein SLN1 in barley largely overcomes the inhibition of growth imposed by low temperature (Fu et al. 2002), suggesting that the DELLA proteins can be an important regulator of thermomorphogenesis **(**Fig. 3**)**. The class I TEOSINTE BRANCHED 1, CYCLOIDEA, PCF (TCP) transcription factors TCP14 and TCP15 control the levels of the DELLA protein RGA, and the expression of growth-related genes in response to an increase in temperature (Ferrero et al. 2019). TCP15 directly targets the gibberellin biosynthesis gene *GA20ox1* to promote cell expansion at warm temperature (Ferrero et al. 2019) (Table 1). TCP5, TCP13, and TCP17 promote PIF4 activity at both transcriptional and post-transcriptional levels under warm temperature conditions (Zhou et al. 2019; Han et al. 2019). DELLAs interact with PIF4, preventing PIF4 from binding to its target promoters, thereby inactivating PIF4 (Feng et al. 2008; Li et al. 2016; Daviere and Achard 2016). Warm temperatures enhance accumulation of the chaperone GIGANTEA (GI), and GI stabilizes the DELLA protein RGA under long day conditions, thereby attenuating PIF4-mediated thermomorphogenesis (Park et al. 2020). In contrast, under short days, GI accumulation is reduced and RGA is readily degraded through the GA signaling pathway, promoting thermomorphogenic growth (Park et al. 2020). In addition, DELLAs also interact with and inactivate ARF6 to fine-tune plant growth under warm temperature conditions (Oh et al. 2014).

### Ethylene and thermomorphogenesis

**Fig. 4:**
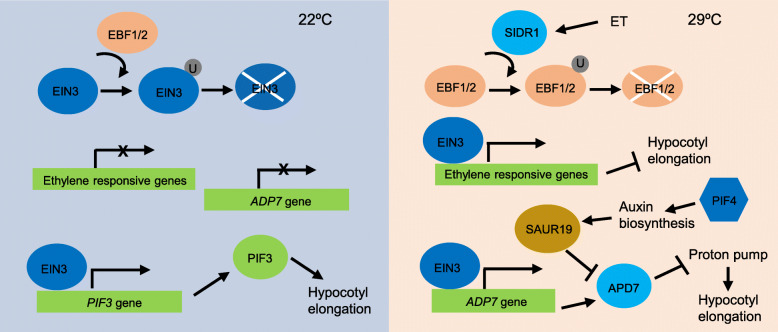
Regulation of plant thermomorphogenesis by the ethylene signaling pathway. Under ambient temperature (22 °C) conditions, when ET contents is low, EIN3 is subjected to EBF1/2-mediated degradation. Low level of EIN3 induces the expression of *PIF3*, enabling hypocotyl growth. Under warm temperature conditions (29 °C), EBF1/2 is ubiquitinated and degraded by SIDR1, releasing the inhibitory effects of EBF1/2 on EIN3. EIN3 upregulates ethylene responsive genes to inhibit hypocotyl growth. EIN3 also upregulates the expression of *APD7*, and APD7 represses the proton pump activity, therefore inhibiting cell expansion. On the other hand, PIF4-mediated auxin signaling represses APD7 activity through SAUR19 to promote hypocotyl elongation. APD7, Arabidopsis PP2C Clade D7; EBF1/2, EIN3-binding F-box protein 1/2; EIN3, ethylene insensitive 3; ET, ethylene; PIF3/4, phytochrome interacting factor 3/4; SIDR1, salt- and drought-induced ring finger 1

Ethylene, a gas hormone synthesized by plants, is widely involved in seed germination, growth, development, and biological and abiotic stress responses (Dubois et al. 2018). Ethylene-mediated hypocotyl growth is contingent on temperature contexts in that ethylene promotes hypocotyl growth at 22 °C but represses it at 28 °C (Fig. 4) (Jin et al. 2018; Kim et al. 2021). Ethylene suppresses hypocotyl growth during skotomorphogenesis, which is mediated by several ethylene-responsive regulators (Sun et al. 2015). Notably, these regulators are involved in the ethylene mediated repression of hypocotyl growth under warm environment (Kim et al. 2021). ERF95 and ERF97 are two ethylene-responsive factors, which are directly regulated by ethylene insensitive 3 (EIN3). Warm temperatures induce the degradation of negative regulators EIN3-binding F-box protein 1/2 (EBF1/2) via the E3 ubiquitin ligase salt- and drought-induced ring finger 1 (SIDR1), therefore leading to more ethylene responses and inhibition of hypocotyl growth (Hao et al. 2021) **(**Fig. 4**)**. Auxin biosynthesis is stimulated by PIF4 and the thermoresponsive PIF4-mediated pathways reinforce the induction of thermomorphogenic growth (Franklin et al. 2011). The ethylene-mediated repression of hypocotyl thermomorphogenesis is compromised in *PIF4*-deficient mutants, suggesting that PIF4 is critical for the reversed action of ethylene under warm temperature conditions. It is known that auxin effects on hypocotyl growth is exerted by cell elongation through SAUR19 (Spartz et al. 2014). Chromatin immunoprecipitation assays revealed that EIN3 directly binds to promoter of Arabidopsis PP2C Clade D7 (APD7), encoding a phosphatase that inhibits H(+)-ATPases (Chang et al. 2013) **(**Fig. 4**)**. At 22 °C, auxin level is relatively low, and thus ethylene promotes hypocotyl growth through the PIF3 pathway (Ma et al. 2018). In contrast, under warm temperature conditions, when auxin is highly accumulated via the PIF4-mediated pathway, the repressive action of the EIN3-APD7 module on the auxin-induced hypocotyl cell expansion is more evident (Kim et al. 2021).

### JA and thermomorphogenesis

The jasmonates (JAs), including jasmonic acid and its derivatives, and salicylic acid (SA) are two important plant hormones modulating inducible defenses (Thaler et al. 2012). JA biosynthesis and its signaling pathway have been well characterized (Howe et al. 2018). JAs are synthesized from α-linolenic acid (α-LeA/18:3) via the octadecanoid pathway, and converted to the bioactive type JA-Isoleucine (JA-Ile), which is regulated by JAR1 (Jasmonate resistant 1) (Huang et al. 2017). In the JA metabolism pathway, three ER localized cytochrome P450 CYP94 family oxidases generate the JA derivatives 12-hydroxy JA-Ile (12OH-JA-Ile), which further forms the 12-hydroxy JA (12OD-JA) after cleaving the Ile group by amido-hydrolases IAR3 and ILL6 (Wasternack and Feussner 2018). Jasmonate-induced oxygenase (JOX)/Jasmonic acid oxidase (JAO) can catalyze the oxidation of JA to 12OH-JA (Heitz et al. 2019; Caarls et al. 2017). Subsequently, 12OH-JA can be sulfated by the sulfotransferase ST2A to form 12-hydroxy jasmonoyl sulfate (12HSO_4_-JA) (Fernandez-Milmanda et al. 2020). JA-Ile is perceived by the receptor complex containing COI1 (Coronatine insensitive 1) and JAZ (Jasmonate ZIM-Domain). The JAZ proteins inhibit transcript factors, such as MYC2, and are subjected to 26S proteasome-mediated degradation after JA treatment (Howe et al. 2018).

Recently, Zhu et al. reported that warm temperature-triggered hypocotyl elongation was largely repressed by MeJA treatment at 28 °C in a concentration-dependent manner in Arabidopsis and wheat (Zhu et al. 2021). Furthermore, the expression of *JOXs* and *ST2A*, genes encoding enzymes involved in the regulation catabolic processes leading to 12HSO4-JA, was significantly up-regulated at warm temperatures (Table 1). In contrast, the Arabidopsis JA deficient *allene oxide synthase* (*aos*) mutant displays a slight longer hypocotyl compared with wild type (Zhu et al. 2021). Therefore, JA content is negatively related to hypocotyl elongation at warm temperatures.

### Conclusion remarks and future perspectives

Plants in nature experience a wide range of daily environmental temperatures. Global warming of 1.5 °C and 2 °C will be exceeded during the twenty-first century unless deep reductions in carbon dioxide (CO2) and other greenhouse gas emissions occur in the coming decades (IPCC, 2021). As a consequence, worldwide cereal production is estimated to have a loss of 6–7% per 1 °C increase in average seasonal temperature and associated extreme temperature conditions (Lesk et al. 2016). A further understanding on how plants perceive and respond to warm temperature is important for designing resilient crops with climate changes in future. However, most of the current research was done with the model plant Arabidopsis. Since different species may have different harmonized growth temperature (Lam Dai et al. 2018; Zhao et al. 2017; Peng et al. 2004), therefore, more work on other crops such as rice, wheat, soybean, should be carried out in future. There are natural variations in *ELF3* in thermomorphogenic responses in Arabidopsis (Raschke et al., 2015). Identification of natural variations in key thermo-regulators in crop plants may help to develop temperature resilient crops through marker-assisted breeding.

Warm temperatures promote growth and accelerate flowering, which helps plants to escape quickly from the soil surface with higher temperature and complete their lifecycle in time (Casal and Balasubramanian 2019). Several thermosensors have been uncovered in plants. Although temperature signals should be quite different from chemical signals, whether there are also membrane-localized thermosensors existed in plants is an open question. Previous studies have shown that phytohormones such as auxin, GAs, BR and ethylene are important for promoting plant growth (Casal and Balasubramanian 2019; Nieto et al. 2015), while JAs inhibit plant growth under warm temperature conditions (Zhu et al. 2021). However, whether other phytohormones are also involved in thermomorphogenesis is not yet reported. ABA-INSENSITIVE3 (ABI3), ABI5, and DELLAs positively activated the expression of SOMNUS (SOM) and other high-temperature-inducible genes to inhibit seed germination (Lim et al. 2013). A recent study showed that ABA interacts synergistically with BR to regulate plant growth and adaptation (Li et al. 2021). These results suggest that it is possible that ABA may also regulate plant growth under warm temperature conditions.

The crosstalk between different plant hormones in plant growth has been reported, either synergistically or antagonistically (Depuydt and Hardtke 2011). The biosynthetic and signaling pathways of auxin and BR are interconnected during thermomorphogenesis (Martinez et al. 2018; Ibanez et al. 2018), whether other plant hormones also have crosstalks under warm temperature conditions awaits future investigation.

## Data Availability

Not applicable.
